# 

**Published:** 1892-10

**Authors:** 


### TABLE OF CONTENTS.

**ORIGINAL COMMUNICATIONS.** PAGE

A Contribution to the Minute Anatomy of the Cementum. By C. HEITZMANN, M.D., and F. A. ROY, M.D., D.D.S. . . . . . . . . . . . . . 709

The Hygiene of the Mouth. By DR. CHARLES E. FRANCIS, New York . . 724

Congenital Cleft Palate and its Treatment. By P. W. MORIARTY, D.M.D., Boston . . . . . . . . . . . . . . . . . . . . . . . . . . . 731

Crown- and Bridge-Work. (*Continued.*) By DR. C. M. RICHMOND, New York . . . . . . . . . . . . . . . . . . . . . . . . . . . . 738

Partial Suppurating Pulpitis—Origin of Certain Alveolar Abscesses in Living Teeth. By ARTHUR C. HUGENSCHMIDT, M.D., D.D.S., Paris, France . . . . . . . . . . . . . . . . . . . . . . . . . . . 741

Gold added to Copper Alloy. By J. E. REGISTER, D.D.S., Dover, Del. . . 745

**REPORTS OF SOCIETY MEETINGS.**

New York Odontological Society . . . . . . . . . . . . . . . . . . 746

American Academy of Dental Science . . . . . . . . . . . . . . . . 747

American Dental Association . . . . . . . . . . . . . . . . . . . 756

**EDITORIAL.**

Honor Those who deserve It . . . . . . . . . . . . . . . . . . . 764

The Relation of Dentistry to Medicine . . . . . . . . . . . . . . . 767

Correction . . . . . . . . . . . . . . . . . . . . . . . . . . . 770

**BIBLIOGRAPHY** . . . . . . . . . . . . . . . . . . . . . . . . 770

**OBITUARY.**

Dr. O. F. Harris . . . . . . . . . . . . . . . . . . . . . . . . 771

Dr. Horatio Leseur . . . . . . . . . . . . . . . . . . . . . . . 772

**FOREIGN CORRESPONDENCE.**

Correspondence and Reply of Dr. W. Mitchell . . . . . . . . . . . . 772

**CURRENT NEWS.**

The World's Congress Auxiliary of the World's Columbian Exposition . . 776

Missouri State Dental Association . . . . . . . . . . . . . . . . 780

Recent Patents . . . . . . . . . . . . . . . . . . . . . . . . . 781

Connecticut Valley Dental Society . . . . . . . . . . . . . . . . . 782

American Academy of Dental Science . . . . . . . . . . . . . . . . 782

Officers of the Massachusetts Dental Society, 1892–93 . . . . . . . . . 782

New England Dental Society . . . . . . . . . . . . . . . . . . . 783

**SELECTIONS.**

Europhen in Minor Surgery . . . . . . . . . . . . . . . . . . . . 783

